# The dual impact of GBA1 in disease: from germline mutations in neurological disorders to alterations in cancer

**DOI:** 10.1038/s41420-026-03046-6

**Published:** 2026-03-19

**Authors:** Valentina Fantini, Giulia Di Rauso, Valentina Fioravanti, Alessia Ciarrocchi, Francesco Cavallieri, Valentina Sancisi

**Affiliations:** 1 Laboratory of Translational Research, Azienda USL - IRCCS di Reggio Emilia, Reggio Emilia, Italy; 2 Neurology Unit, Neuromotor & Rehabilitation Department, Azienda USL-IRCCS of Reggio Emilia, Reggio Emilia, Italy; 3 Clinical and Experimental Medicine PhD Program, University of Modena and Reggio Emilia, Modena, Italy

**Keywords:** Cancer genetics, Molecular neuroscience

## Abstract

The *GBA1* gene encodes the enzyme glucocerebrosidase, which is responsible for lysosomal degradation of the glycosphingolipid glucosylceramide. Biallelic mutations in *GBA1* are causative for Gaucher disease, whereas either monoallelic or biallelic mutations are a risk factor for Parkinson’s disease. *GBA1* mutations, beside reducing enzymatic activity and leading to substrate accumulation, influence a number of molecular and cellular pathways, including lipid homeostasis, endosome-lysosome pathway, endoplasmic reticulum to Golgi protein trafficking, autophagy and mitophagy. Given the critical role of GBA1 in these key pathways for cellular homeostasis, it can be expected that alterations in this enzyme may influence also cancer development and/or pathology, keeping in mind that Gaucher disease is associated with an increased risk of cancer development. Notably, a large fraction of patients affected by different cancer types carry an amplification of the long arm of chromosome 1, that includes the *GBA1* gene. Furthermore, GBA1 expression is elevated in different cancer tissues, compared with healthy counterparts and associated with outcome in some cases. In this perspective, we narratively review the main evidence supporting a role for *GBA1* in influencing tumorigenesis and we present our analyses on *GBA1* amplification and expression throughout different cancer types. Taken together, these data suggest that the presence of a *GBA1* germline mutation or a somatic amplification may influence cancer pathogenesis and/or response to therapies through context-dependent mechanisms that are still to be characterized.

Several studies demonstrated that the presence of pathogenic germline variants in oncogenes, tumor suppressors or in DNA damage response genes may not only influence the risk of cancer onset but also modify cancer pathology and response to therapy [1–3]. However, how germline non-pathogenic variants or mutations associated with rare diseases influence cancer pathogenesis is largely still to be investigated.

The *GBA1* gene encodes for the lysosomal enzyme glucocerebrosidase (GCase), which is responsible for degradation of glucosylceramide (GlcCer), a membrane glycosphingolipid. Biallelic mutations in *GBA1* are causative for Gaucher disease (GD), the most frequent lysosomal storage disorder, whereas either monoallelic or biallelic mutations in this gene are the most common genetic risk factor for Parkinson’s disease (PD) [4, 5]. PD is a common neurodegenerative disease, characterized by progressive degeneration of dopaminergic neurons, leading to a broad range of motor and non-motor symptoms [6]. More than 300 different *GBA1* mutations have been shown to be causative for GD, being the point mutations N370S and L444P the most common [7]. GD exhibits a wide range of clinical presentations, spanning from asymptomatic forms to severe organ damage and neurological involvement. From a molecular point of view, in GD, *GBA1* mutations lead to decreased enzymatic activity with consequent accumulation of its substrate, GlcCer, and other sphingolipids, that exert toxic effects. All GD forms are characterized by the presence of Gaucher cells, lipid-engulfed macrophages showing a typical morphology and altered functionality. Gaucher cells infiltration in different organs, including bone marrow, spleen and liver is considered the main pathogenic event in GD [8]. *GBA1* variants can be classified based on the severity of the GD phenotype and in particular on the presence of neurological symptoms, being GD1 typically characterized by only visceral involvement, GD2 by visceral and neurological involvement and GD3 by variable presentation [8]. Carriers of *GBA1* mutations, both in heterozygosis and homozygosis, show a 5-30-fold increased risk of developing PD. Notably, carriers of severe *GBA1* mutations, such as L444P, show an increased risk, compared to carriers of mild mutations, like N370S. However, it remains uncertain whether the risk differs between biallelic carriers (i.e., GD patients) and heterozygotes, and even carriers of certain variants (“risk variants”) that are not pathogenic for GD have an increased risk of PD [9–12].

These findings suggest that different mechanisms may be implicated in GD and in *GBA1*-related PD. Importantly, GlcCer accumulation in the brain of *GBA1*-mutated PD patients has never been reported, suggesting that substrate accumulation may not be the molecular link between *GBA1* mutations and PD development [13]. Indeed, *GBA1* mutations have been shown to interfere with a number of biological pathways through different mechanisms (Fig. 1). The contribution of each of these mechanisms to GD and PD pathogenesis is still to be completely elucidated. Mutant GCase isoforms do not fold correctly in the endoplasmic reticulum (ER), leading to protein accumulation and activation of unfolded protein response (UPR), that can cause neuron damage and death [14–16]. The main molecular hallmark of PD is the accumulation of α-synuclein aggregates in dopaminergic neurons, called Lewy bodies. Mutated GCase enzymes lead to α-synuclein accumulation, while α-synuclein aggregates interfere with trafficking of GCase from the ER to the lysosome, in a vicious pathogenic loop [17–19]. Dysfunction of GCase can result not only in the intracellular accumulation of its substrate GlcCer, but also in its conversion in glucosylsphingosine (GlcSph) [20]. These sphingolipids may affect the fluidity of lysosome membranes and promote the formation of α-synuclein aggregates [21–23]. Additionally, cellular and animal models of GCase deficiency show impairment in the autophagy-lysosome pathway, leading to decreased α-synuclein clearance [24, 25]. In the *GBA1* mutant context, also mitophagy is defective, leading to dysfunctional mitochondria accumulation, with reduction of ATP production and increase in reactive oxygen species (ROS) and free radicals [26–30]. Importantly, altered autophagy and mitophagy dysfunction are also associated with mutations in other genes causative for genetic PD, such as *PINK1*, *parkin*, *LRKK2* and *DJ-1* [31]. Furthermore, *GBA1* germline mutations are associated to a general picture of immune dysregulation. Gaucher cells display properties of anti-inflammatory alternatively activated macrophages, such as the expression of CD163, CCL18, and interleukin-1 receptor antagonist [32]. In Gaucher macrophages, impaired autophagy activates the inflammasome, leading to altered cytokine secretion, including IL-1β and IL-6 [33]. In neuronopathic GD, a cytotoxic role for neuroinflammation and in particular of activated microglia is well documented [34, 35]. Furthermore, Type II NKT cells against sphingolipids accumulated in GD patients have been identified. These cells are increased in a GD mouse model and in GD patients, holding the capacity to stimulate B cells and the production of anti-lipids antibodies, thus contributing to chronic inflammatory phenotype in GD [36]. On the other hand, also in *GBA1*-related PD, broad alterations in lymphocyte counts, proinflammatory factors and chemokines, as well as serum neurosteroids have been observed [37–41].

**Fig. 1:**
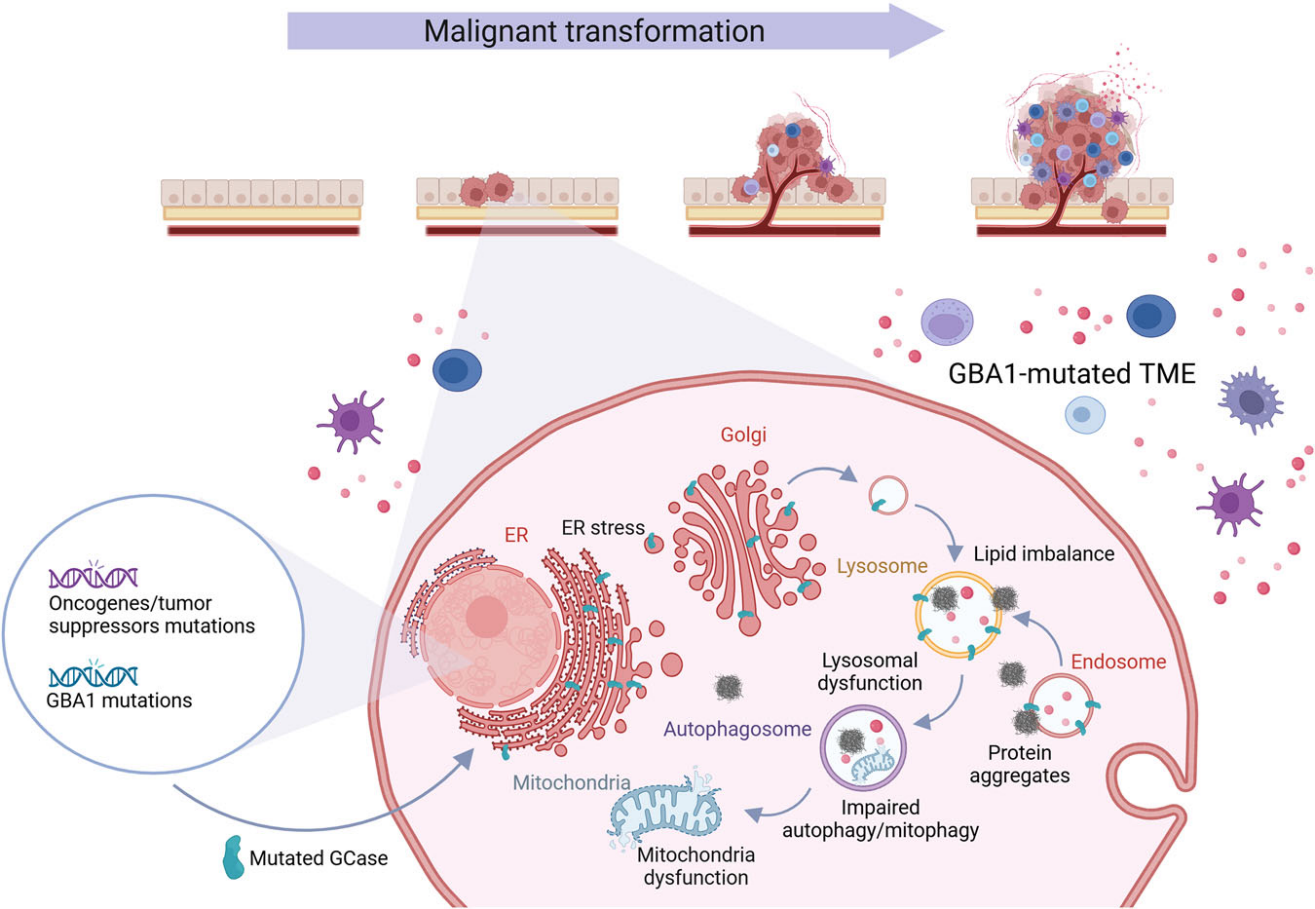
Overview of the pathways that are affected by GBA1 alterations. During malignant transformation, neoplastic cells may grow in a GBA1-altered background due to somatic Chr1q amplifications or germline *GBA1* point mutations. Altered GBA1 function has been described to influence different pathways in the context of neurological diseases, including lipid homeostasis, endosome-lysosome pathway, endoplasmic reticulum (ER) to Golgi protein trafficking, autophagy and mitophagy. Alteration in these pathways due to the presence of *GBA1* germline mutations or somatic amplifications may also modify cancer pathogenesis. Additionally, patients carrying a germline *GBA1* mutation harbor the mutation also in the tumor microenvironment cell populations, with the potential to modify both cancer pathogenesis and response to therapies. Created in BioRender. Sancisi, V. (2025) https://BioRender.com/lmb95ok.

Overall, these findings highlight the complexity of the molecular and cellular alterations induced by *GBA1* mutations, relying both on loss-of-function and gain-of-function mechanisms. Given the critical role of the GCase enzyme in a number of key pathways for cellular homeostasis, it can be expected that alterations in the biology of this enzyme may influence also cancer development and/or pathology (Fig. 1).

Indeed, it is well established that GD is associated with an increased risk of cancer development [42–46]. The overall risk varies depending on the study and population considered, ranging from 0.79 to 3.6 [46]. The most prevalent malignancies are hematological cancers and in particular multiple myeloma [44–46]. Among solid malignancies the most frequently reported are melanoma and hepatocellular carcinoma [44–46]. A minority of studies reported a lack of association between GD and the overall cancer risk [47] or reported only the association with hematological malignancies and not with solid tumors [48]. Conversely, the association between PD and cancer is less clear. Idiopathic PD is generally associated with a lower risk of cancer development, except for melanoma and brain cancer [49–51]. Notably, carriers of the *LRRK2* G2019S mutation appear to have a higher risk of developing certain types of cancer compared to idiopathic PD patients, as suggested by previous studies [50]. No studies have been published yet on the risk of cancer development in the *GBA1*-PD population. Overall, the increased risk of cancer development described in GD patients may suggest a potential involvement of GBA1 mutations in the pathogenesis of at least some cancer types.

To start dissecting the possible role of *GBA1* in cancer pathogenesis, we assessed the presence of somatic alterations in this gene in a large cohort of cancer patients derived from The Cancer Genome Atlas (TCGA) and comprising 33 cancer types. We found that a large fraction of patients affected by different cancer types carry a somatic amplification of the *GBA1* gene. *GBA1* is localized in the long arm of chromosome 1 (Chr1q), in a genomic locus that is well known to be frequently amplified in many cancer types [52]. The percentage of amplified patients is around 20%-40% in some of the most common malignancies, such as breast cancer, lung adenocarcinoma, melanoma and liver cancer (Fig. 2A).

**Fig. 2:**
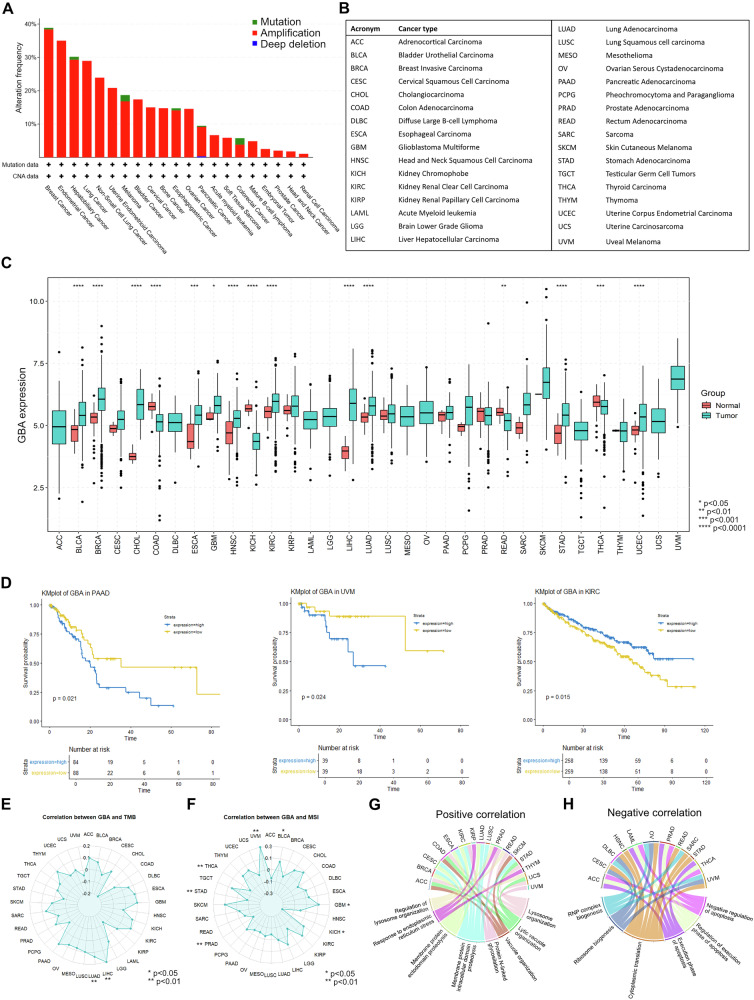
GBA1 alterations in cancer. **A** Alteration frequency of point mutation, amplification or deletion in the *GBA1* gene in different cancer types. The data are from the pan-cancer TCGA cohort [62]. **B** Acronyms of cancer types shown in (**C**)–(**H**). **C** GBA1 expression level in tumor and normal tissue, in different cancer types. **D** Kaplan-Meier curve representing overall survival probability in patients affected by PAAD, UVM or KIRC, presenting high or low GBA1 expression. **E**, **F** Correlation between GBA1 expression and tumor mutational burden (TMB) (**E**) or microsatellite instability (MSI) (**F**) in different cancer types. **G**, **H** Analysis of the enriched GO pathways positively (**G**) or negatively (**H**) associated with GBA1 expression. Plots C-F are made using TCGAplot R tools [63]. **G** and **H** are made using TCGAplot R tools, with some modifications. *p < 0.05, **p < 0.01, ***p < 0.001, ****p < 0.0001.

To assess whether GBA1 elevated expression could be pro-oncogenic, we compared GBA1 expression levels between cancer tissues and healthy counterpart, finding a significant overexpression of GBA1 in different cancer types, including breast (BRCA), lung adenocarcinoma (LUAD) and liver hepatocellular carcinoma (LIHC) (Fig. 2B, C). On the contrary, some cancer types displayed a significant downregulation in the expression of this gene, such as colon carcinoma (COAD) and thyroid carcinoma (THCA) (Fig. 2B, C). Furthermore, in some cancer types, higher expression of GBA1 was associated with worse outcome, such as pancreatic adenocarcinoma (PAAD) and uveal melanoma (UVM), whereas in kidney renal clear cell carcinoma (KIRC) was associated with a better outcome (Fig. 2D).

Next, we evaluated the correlation between GBA1 expression and tumor mutational burden (TMB) and microsatellite instability (MSI). Intriguingly, we found a positive correlation with TMB in LUAD and LIHC (Fig. 2E). MSI was positively correlated with GBA1 expression in uveal UVM, glioblastoma multiforme (GBM) and bladder urothelial carcinoma (BLCA) and negatively correlated in kidney chromophobe carcinoma (KICH), prostate adenocarcinoma (PRAD), stomach adenocarcinoma (STAD) and THCA (Fig. 2F). In line with its biological role, GBA1 expression was positively correlated with alterations in pathways involved in lysosome organization, protein glycosylation, ER stress response and proteolysis (Fig. 2G). Conversely, GBA1 expression was negatively correlated with ribosome biogenesis, protein translation and execution of apoptosis (Fig. 2H). Overall, these correlative findings suggest that GBA1 somatic alterations may impinge on several pathways implicated in cancer development and progression, ultimately influencing cancer pathogenesis in a context-dependent manner.

We found GBA1 expression significantly elevated in liver cancers, such as LIHC and CHOL and positively associated with TMB in LIHC. Hepatocellular carcinoma is also one of the solid tumors showing increased risk in GD patients, suggesting a particularly relevant role of *GBA1* in this cancer type [42, 44]. Intriguingly, two recent publications investigated the role of *GBA1* in liver tumorigenesis with opposing conclusions. Qiu and colleagues showed that GBA1 knockdown is associated with increased cell proliferation, cell invasive properties, tumor growth and metastatization in vivo, while GBA1 overexpression leads to a decrease of these cancer cell features. GBA1 downregulation promotes epithelial-mesenchymal transition (EMT) through activation of the Wnt/β-catenin pathway. Mechanistically, GBA1 knockdown increases plasma membrane GlcCer, which directly interact with the Wnt receptor component LRP6, promoting its phosphorylation and Wnt signaling transduction [53]. Undertaking a completely different approach, Vasquez Salgado and coworkers performed a CRISPR activation screening to identify the genes that are localized on the Chr1q amplification that modify tumor growth in an in vivo model of liver cancer. In this context, overexpression of Gba1, Mrpl9 or Vps72 increased liver tumorigenesis. They showed that overexpression of Gba1 is associated with deregulation of genes implicated in endosome-lysosome regulation [54]. It should be noted that these two studies relied on two completely different in vivo cancer models, since Qiu and collaborators performed orthotopic xenograft experiments in nude mice, while Vazquez Salgado and colleagues used a MYC-driven genetic model of tumorigenesis to screen for genes which promoted tumor formation. Importantly, this latter model is immunocompetent, suggesting that a relevant source of discrepancy between the results of the two studies may be the presence of mouse immune system. Additionally, Qiu et al. observed a decrease of GBA1 levels in human tumor samples by immunohistochemistry, supported by a modest increase in GlcCer. On the contrary, Vazquez Salgado et al. reported an increased GBA1 mRNA level in tumor samples compared with healthy liver. This discrepancy may be due to differences in the composition of the two patients’ cohorts. Indeed, the work of Qiu et al. showed that GBA1 protein levels also differ between patients at different stages and with or without vascular invasion, suggesting that cohorts containing patients with different characteristics may show contradictory results. On the other hands, the results of Vazquez Salgado et al., showing GBA1 overexpression in tumor samples at mRNA level, are in line with the data we showed in Fig. 2C. This confirms the overexpression of GBA1 at mRNA level in tumor samples and may suggest the possibility of discordance between mRNA and protein levels. However, this hypothesis needs to be confirmed through parallel analysis of GBA1 mRNA and protein levels in the same sample set. Overall, further investigation is needed to reconcile these findings and to clarify the role of GBA1 in liver tumorigenesis. Notably, other publications reported an involvement of GBA1 in modulating cancer cell proliferation, EMT and drug resistance in other cancer types [55–58]. It is also well established the correlation between elevated levels of GlcCer and/or of the glucosylceramide synthase enzyme with multidrug resistance [59].

Taken together, these reports and the data we presented suggest that *GBA1* may have a still underestimated role in cancer. The presence of frequent somatic alterations and overexpression in different cancer types may suggest a pro-oncogenic role for this gene. On the other hand, some cancer types show GBA1 downregulation and the few available mechanistic studies reported conflicting results, as in the case of liver cancer. This may implicate a context-dependent modulatory role of GBA1 on cancer pathogenesis, that needs to be clarified by further studies. Additionally, given the involvement of GBA1 in different pathways deeply implicated in cancer pathogenesis, it is possible that somatic or germline alterations in this gene, such as those carried by GD and PD patients, may exerts effects on malignancy insurgence and/or cancer pathogenic properties, including response to treatments (Fig. 1). Notably, an association between other PD-associated genes and cancer has been recently reported, further underlying the relevance of shared pathways between cancer and neurodegenerative disease [60]. In particular, it should be taken in consideration that germline alterations in *GBA1* may influence both cancer cells and the tumor microenvironment. As already mentioned, it has been shown that different immune cell populations display altered numbers and/or functionality, as a consequence of germline *GBA1* mutations in GD patients. In Gaucher cells, autophagy disfunction leads to alterations in inflammatory profile, including increased IL-6 and IL-1β cytokine production [33]. Gaucher cells dysregulation arising from accumulation of lipids may result in impaired immune surveillance, suggesting the possibility that these cells may also influence tumor behavior during malignant transformation, supporting a pro-tumoral microenvironment [45]. Gaucher cells influence on the tumor microenvironment may contribute to the reported increase of cancer incidence in GD patients. The consequences of *GBA1* alterations on the immune milieu may also be relevant for response to anti-cancer immune-therapeutics which are increasingly employed for the treatment of patients affected by different cancer types [61].

In conclusion, based on our literature review and bioinformatic analyses of publicly available cancer databases, we report preliminary evidence suggesting a possible association between *GBA1* gene alterations and cancer. The role of this gene in cancer pathogenesis and the consequences of germline mutations or somatic alterations for cancer patients still need further investigation to be clarified. In this context, we recommend the development of functional and translational studies.

## Data Availability

The gene expression data and clinical data are available in The Cancer Genome Atlas (TCGA) database, downloaded from GDC Data Portal (Trascriptome Profiling and Clinical Data, using TCGAbiolinks R tools). Mutational Data are available on cBioPortal.
